# Selection and validation of reference genes for gene expression analysis in apomictic and sexual *Cenchrus ciliaris*

**DOI:** 10.1186/1756-0500-6-397

**Published:** 2013-10-02

**Authors:** Bindu Simon, Joann A Conner, Peggy Ozias-Akins

**Affiliations:** 1 Department of Horticulture, The University of Georgia Tifton Campus, Tifton, GA 31793, USA; 2 Current Address: Burnett School of Biomedical Sciences, University of Central Florida, Florida 32826, USA

**Keywords:** Apomixis, Apospory, Reference genes, qRT-PCR, Buffelgrass, *Cenchrus ciliaris*

## Abstract

**Background:**

Apomixis is a naturally occurring asexual mode of seed reproduction resulting in offspring genetically identical to the maternal plant. Identifying differential gene expression patterns between apomictic and sexual plants is valuable to help deconstruct the trait. Quantitative RT-PCR (qRT-PCR) is a popular method for analyzing gene expression. Normalizing gene expression data using proper reference genes which show stable expression under investigated conditions is critical in qRT-PCR analysis. We used qRT-PCR to validate expression and stability of six potential reference genes (EF1alpha, EIF4A, UBCE, GAPDH, ACT2 and TUBA) in vegetative and reproductive tissues of B-2S and B-12-9 accessions of *C. ciliaris*.

**Findings:**

Among tissue types evaluated, EF1alpha showed the highest level of expression while TUBA showed the lowest. When all tissue types were evaluated and compared between genotypes, EIF4A was the most stable reference gene. Gene expression stability for specific ovary stages of B-2S and B-12-9 was also determined. Except for TUBA, all other tested reference genes could be used for any stage-specific ovary tissue normalization, irrespective of the mode of reproduction.

**Conclusion:**

Our gene expression stability assay using six reference genes, in sexual and apomictic accessions of *C. ciliaris*, suggests that EIF4A is the most stable gene across all tissue types analyzed. All other tested reference genes, with the exception of TUBA, could be used for gene expression comparison studies between sexual and apomictic ovaries over multiple developmental stages. This reference gene validation data in *C. ciliaris* will serve as an important base for future apomixis-related transcriptome data validation.

## Background

Sexual and asexual modes of reproduction occur in flowering plants. The sexual reproduction pathway is highly regulated and results in the production of seed *via* fusion of male and female gametes. Apomixis is a natural form of asexual reproduction through seeds. There are two basic forms of apomixis, sporophytic and gametophytic. Sporophytic apomixis (or adventitious embryony) results in embryos that develop directly from nongenerative cells of the ovule. Gametophytic apomixis involves three steps: formation of a meiotically unreduced egg cell (apomeiosis), parthenogenetic development of this egg cell without fertilization, and formation of functional endosperm with (pseudogamy) or without (autonomous) fertilization of the central cell of the ovule [1,2]. Apomeiosis in gametophytic apomixis consists of two forms: apospory and diplospory. In apospory, the embryo sacs develop from nucellar cells, while in diplospory the generative cell undergoes mitosis to form an embryo sac. In both apospory and diplospory, a chromosomally unreduced cell gives rise to the megagametophyte, in which the unreduced egg cell parthenogenetically develops into an embryo that is genetically identical to the maternal plant [1,2]. Hence the successful introgression of apomixis into crop plants would greatly facilitate maintenance of hybrid vigor over successive generations and also reduce costs associated with hybrid seed production [3]. Apomixis is a common mode of asexual reproduction in numerous families but is most frequent in the eudicot families Rosaceae and Asteraceae and in the monocot family Poaceae [4,5]. The most common hypothesis behind apomictic reproduction is that it is evolved from the sexual pathway, possibly by deregulation in the timing of expression of sexual reproductive genes [6]. The global regulatory effects of polyploidy and hybridity have also been proposed to be the possible triggers for conversion of sexual to apomictic forms of reproduction [1,7]. Genetic data from several species now indicate that at least two genes, one for apomeiosis and one for parthenogenesis [2], are required for apomixis, although lack of recombination in some species manifests as monogenic inheritance of the trait.

*Cenchrus ciliaris* (buffelgrass, syn. *Pennisetum ciliare*) is found in tropical and southern Africa, the Mediterranean region, India and Pakistan. Most accessions of *C. ciliaris* are tetraploid with 2n = 4 × = 36 [8]. Apospory in buffelgrass is conferred by an apospory-specific genomic region (ASGR) that is hemizygous in nature and recalcitrant for recombination [9-14]. *C. ciliaris* displays the 4-nucleate *Panicum*-type aposporous embryo sac pheno-type [15,16]. A sexual genotype discovered in *C. ciliaris* was crossed as the female parent to apomictic genotypes producing progeny in three phenotypic classes, obligate sexual, obligate apomictic and facultative apomicts [14,17,18]. Regions of the ASGR in *C. ciliaris* have been studied *via* partial sequencing of ASGR-linked BAC (Bacterial Artificial Chromosome) clones, leading to the identification of various genes with putative transcription factor or signaling-related functions [19].

Differential gene expression studies between sexual and apomictic plants enables comparison between apomictic and sexual pathways. In *Boechera*, advances in cell isolation methods, along with next generation sequencing technology, have allowed global comparisons of gene expression patterns between sexual and apomictic reproductive tissues [20-22]. Quantitative real-time, reverse-transcriptase PCR (qRT-PCR) is one of the most extensively used methods to analyze and validate transcript expression profiles among different species, treatments or developmental stages due to its high sensitivity, specificity and broad quantification range [20,23]. However accurate normalization steps should be followed to obtain reliable quantification of gene expression levels *via* qRT-PCR. The purpose of normalization is to correct any non-biological variability during the experimental procedure [24,25]. Among the various normalization approaches, the use of internal controls or reference genes has become the method of choice [26,27]. The success of using reference genes for proper normalization in qRT-PCR is highly dependent on the choice of the appropriate reference gene: its expression should be relatively constant across tissues and should not be significantly altered by the experimental conditions [28,29]. Since reference genes often are housekeeping genes required for cellular survival, it is assumed that they are stably expressed across all tissues and/or treatments which often is not the case [24,30]. Different statistical software like GeNorm [31] and NormFinder [32] are available to test expression stability of reference genes. The most commonly used reference genes for various qRT-PCR analyses are actin, glyceraldehyde-3-phosphate dehydrogenase, ribosomal genes, cyclophilin, elongation factor 1 alpha, adenine phosphoribosyl transferase and tubulin [33-38].

With respect to testing reference genes for apomictic gene expression analysis, two plants, *Boechera* and *Brachiaria brizantha,* have been used to validate reference genes in their respective apomictic and sexual accessions in different tissues and developmental stages [20,39]. In *Boechera*, EF1alpha (Elongation factor 1 alpha) and ACT2 (Actin 2), were among the stable genes detected [20] and in *Brachiaria brizantha*, UBCE (ubiquitin conjugating enzyme), EIF4A (eukaryotic initiation factor 4A) and EF1 alpha were the most stable genes in both apomictic and sexual plants [39]. TUBA (tubulin alpha) has been used as an internal control gene to normalize the qRT-PCR data for *Pennisetum glaucum* interspecific hybrids [40]*.* In the present paper, we selected six reference genes – EF1alpha, EIF4A, ACT2, UBCE (chosen based on their stability in *Boechera* and *Brachiaria brizantha*), GAPDH (glyceraldehyde-3-phosphate dehydrogenase, the most commonly used reference gene in different systems), and TUBA (reported as unstable in *Boechera* and *Brachiaria brizantha*[20,39] but used as an internal control for *Pennisetum glaucum* interspecific hybrids [40]). All the six reference genes were tested for gene expression stability in both sexual and apomictic *C. ciliaris* in multiple tissues but specifically in reproductive tissues encompassing four developmental stages for ovary and three developmental stages for anthers.

## Methods

### Plant materials and sample collection for qRT-PCR

Two genotypes of *C. ciliaris* (buffelgrass syn. *Pennisetum ciliare*) used in this study (obligate sexual, B-2S, and obligate aposporous, B-12-9) were maintained in the greenhouse with a temperature ranging from 24°C to 30°C. The plants were maintained by vegetative propagation as described in Roche *et al.*[14].

Leaves and roots were collected from greenhouse-grown B-2S and B-12-9 plants, frozen in liquid nitrogen and stored at −80°C prior to total RNA extraction. To collect reproductive tissues, young inflorescences from B-12-9 and B-2S grown in the greenhouse were covered with pollination bags to prevent cross-pollination during the flowering season. Four developmental stages were collected for ovary samples based on anther developmental stages: stage I, early premeiotic; stage II, tetrad; stage III, DOP (the head contained one or two florets with anthers emerged, although tissue was collected from florets in which anthers had not yet emerged); and stage IV, DOP + 5 days (pollinated and seed were developing). Three different stages were collected for anthers: stage I, early premeiotic; stage II, tetrad; stage III, DOP (tissue was collected from anthers that had not yet emerged). The developmental stages I and II were determined by carbol fuchsin staining of anther squashes [41]. Staged inflorescence segments were initially stored in RNALater (Ambion) at −20°C and later ovary and anther samples were dissected and stored in RLT lysis buffer (Qiagen) at −80°C. At least two biological replicates were collected for all samples.

### Total RNA extraction and cDNA synthesis

Total RNA was extracted using the RNeasy plant mini kit (Qiagen) and then subjected to DNAase treatment (Turbo DNA free DNase, Ambion). The DNase treated RNA was concentrated using RNeasy mini elute columns (Qiagen). The final total RNA samples were checked by a Nanodrop spectrofluorometer (260/280, 260/230 ratios). RNA with 260/280 in the range of 1.9-2.0 and 260/230 > 2 were checked for RNA integrity using an Agilent Bioanalyzer. Only RNA with a RIN score > 7 was used for the experiment. First strand cDNA synthesis used 5 μg of total RNA with oligo-dT and superscript II enzyme (Invitrogen), according to the manufacturer’s instructions. The generated cDNAs were quantitated in triplicate using ribogreen [42], diluted to 3 ng/μl and stored in aliquots to avoid freeze thaw cycles.

### Sequencing of conserved reference gene domains from B-2S and B-12-9

EST sequences from conserved regions of each reference gene were used to design the first set of primers (Table 1) using Primer3 (v. 0.4.0) software (http://frodo.wi.mit.edu/, [43]). Using the conserved region primers from Table 1, fragments specific to each reference gene were amplified from both B-2S and B-12-9 leaf cDNA, cloned into pCR®-Blunt II-TOPO® vector (Zero Blunt®TOPO® PCR Cloning Kit, Invitrogen) and sequenced at the Georgia Genomics Facility (Athens, GA). At least 17 sequences per genotype from each reference gene were aligned using clustalW2 (http://www.ebi.ac.uk/Tools/msa/clustalw2/).

**Table 1:** Candidate reference gene description and details of the first set of primer sequences designed from the conserved regions of the specific reference genes

**Gene name/description**	**Gene function**	**NCBI accession**^**1**^	**Primers-(5′-3′)**	**Amplicon size (bp)**
			**forward/reverse**	
EF1alpha/elongation			AGGAACTTGGGCTCCTTCTC	225
Factor 1alpha	Translation	EB672663.1	/TCTCAAGCGTGGTTATGTGG	
EIF4A/eukaryotic			CTGGCAGCTCCTCAATCAC	
Initiation factor 4A	Translation	EB659100.1	/TTTGCCACTCACGGTGACAT	301
ACT2/actin2	Cytoskeleton	EB670295.1	ATCGTACTCCGCCTTTGAGA	425
			/ACTACGAGCAGGAGCTGGAG	
UBCE/ubiquitin	Protein	EB661936.1	GGGCTGTCGACTCGTACTTT	433
Conjugating enzyme	Aggregation		/CTGCGGAAGGAGTTTGTCAT	
GAPDH/glyceraldehyde-3-phosphate dehydrogenase	Glycolysis	EB653168.1	TCGTACCAGGAGACGAGCTT	404
			/ATCACTGCCACCCAGAAGAC	
TUBA/tubulin alpha	Microtubules	EB655254.1	TTCTCCATCATCACCTTCGTC/	560
			TTTGATGGTGCTATCAACGTG	

^1^ NCBI accession id for the *C. ciliaris* ESTs that were used in the primer design.

### Primer design and real-time PCR assay

A set of real-time primers was designed from the aligned region of each of the six reference genes (EF1alpha, EIF4A, UBCE, GAPDH, ACT2 and TUBA) using Primer3, (v. 0.4.0) software [43] and oligo analysis was performed using IDT oligoanalyzer tool (http://www.idtdna.com) with care taken to ensure that the reference gene real-time primer sequences were from the conserved regions of B-2S and B-12-9 and did not encompass any SNPs (single nucleotide polymorphisms). Real-time RT-PCR amplification reactions were performed using SYBR Green detection chemistry and run on 384-well plates with the Light Cycler 4.8 (Roche Applied Science). To estimate PCR efficiency of each reference gene primer pair, standard curves were generated using cDNA samples derived from different organs (leaf, root, anther and ovary) for both B-2S and B-12-9 genotypes. Samples were run in a 2-fold serial dilution range across 5 points with initial concentration of template starting at 0.6 ng. The corresponding PCR efficiencies and the error values were determined by the Light Cycler 4.8 software. All samples to be analyzed were run in the same plate in triplicate. Reactions were prepared in a total volume of 10 μl containing: 2 μl of template, 1 μl of each reference gene primer pair (optimized concentration: 300 nM), 5 μl of 2x FastStart SYBR Green Master (Roche Applied Science) and 2 μl of sterile water. Each PCR analysis also included reverse transcription negative control (RT minus, without reverse transcriptase) to confirm the absence of genomic DNA and a no-template negative control to check for primer-dimer and contamination. Uracil-N-Glycosylase (1 μl per 100 μl of reaction mixture) was also included in the reaction mixture in order to avoid PCR product carryover contamination. The PCR cycling conditions were set as follows: pre-incubation at 37°C for 10 min to activate Uracil-N-Glycosylase, an initial denaturation step of 95°C for 10 min to inactivate Uracil-N-Glycosylase and activate the FastStart Taq DNA polymerase, followed by 45 cycles of denaturation at 95°C for 10s, annealing at 60°C for 10s and extension at 72°C for 10s. The amplification process was followed by a melting curve analysis, ranging from 65°C to 90°C, increasing temperature in steps of 0.2°C every 10s. Cp values were automatically determined using the Light Cycler 4.8 software (absolute quantitation *via* second-derivative method). For every cDNA sample, the mean expression level and standard deviation for each set replicate was calculated (cut-off value for standard deviation was kept as 0.3; only samples below this cut-off were considered). To confirm the reproducibility of the assay and to reconfirm reference gene stability, the experiment was repeated for B-2S and B-12-9 ovary samples. Two biological and three technical replicates were included.

### Gene expression stability analysis

For ranking reference genes based on their stability, GenEx (version 4.1.7, MultiD Analyses) which includes both GeNorm and NormFinder software was used. The Microsoft Excel file with raw expression Cp values for each tested gene in the 18 different samples generated with the Light Cycler 4.8 software was transferred into GenEx software.

## Findings

**Figure 1:**
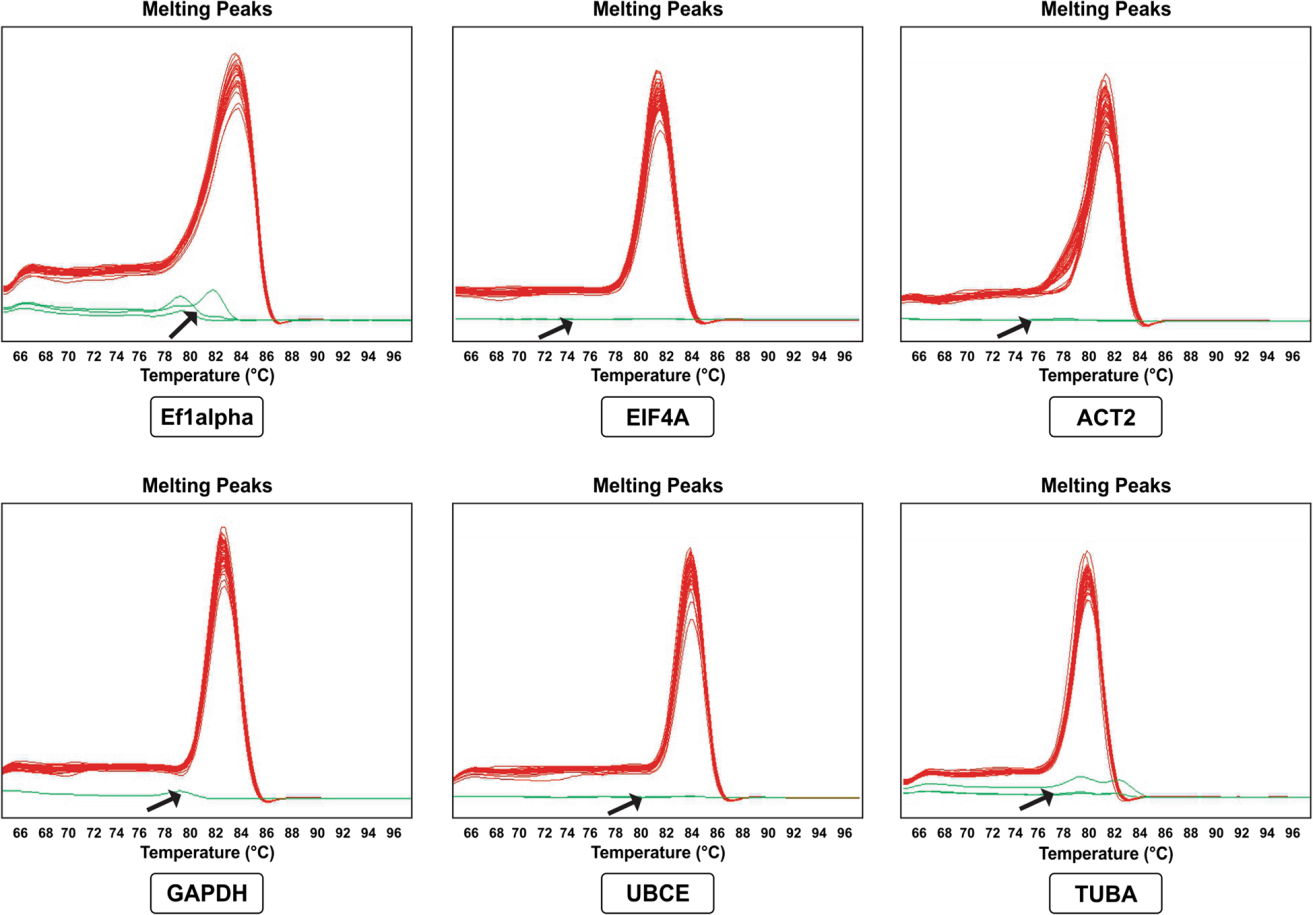
**Specificity of PCR amplification.** Dissociation curves for all six reference genes with a single product peak were obtained using three technical replicates of 18 cDNA samples. The arrow indicates no template control.

Six reference gene sequences (EF1alpha, EIF4A, UBCE, ACT2, GAPDH and TUBA) from other plant species were used in BlastN analysis against the *C. ciliaris* pistil EST library at NCBI where *C. ciliaris* EST sequences with high similarity to all six selected reference genes (E-value = 0.0) were retrieved. High sequence similarity with the specific reference genes from other plant species (E-values below 7e-179) was confirmed by BlastN of the selected *C. ciliaris* EST sequences (Table 1) against the non-redundant database at NCBI. The amplicon length for the first set of primers used to generate B-2S and B-12-9 clones ranged between 225–560 bp whereas amplicon length with real-time RT-PCR primers ranged between 80–106 bp. Primer T_m_ ranged between 59-60°C, and primer lengths varied between 20–26 bp. All real-time RT-PCR primers specific for the reference genes showed acceptable amplification efficiencies (Table 2), and dissociation curves showed a single PCR product for each (Figure 1).

**Table 2:** Primer sequences used for qRT-PCR analysis

**Gene name**	**Primers-(5′-3′) forward/reverse**	**Amplicon size (bp)**	**PCR Efficiency ± SD**^**1**^
EF1alpha	GTGGTTCATGATGATGACCTGGGA/	82	1.90 ± 0.03
	TGGTTATGTGGCCTCCAACTCCAA		
EIF4A	TGGTGATGAGCACACGGGATGAA/	80	1.90 ± 0.05
	TCACGGTGACATGGACCAGAACACTA		
ACT2	CCTTCCTGATATCCACATCACA/	103	1.85 ± 0.03
	CCTGAGGTCCTCTTCCAACC		
UBCE	TGTCATGGCATCGAAGCGTATCCT/	106	1.87 ± 0.03
	TTGCCAATGAAACATGTCCTCGCC		
GAPDH	TGTCACCAGTGAAGTCCGTGGAAA/	94	1.95 ± 0.04
	AAGAAGGCTATCAAGGCTGCGTCT		
TUBA	CTGCAGAATTCAGGTTTGATGGTGC/	80	2.05 ± 0.05
	GATACGTGGGTATGGAACAAGGTTGG		

^1^*SD*, standard deviation; efficiency values for genes were calculated using the standard curve method of Light Cycler 4.8 software (absolute quantitation *via* second derivative method). An efficiency of 2 denotes 100%. All the error values for standard curves were below 0.02.

**Figure 2:**
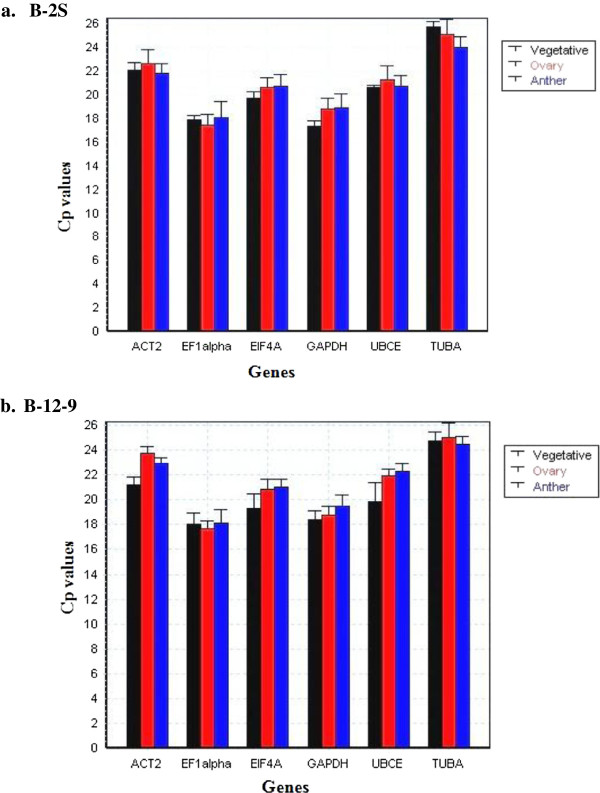
**Cp distribution graph.** The plot shows the Cp distribution of each candidate reference gene for the different samples (Vegetative-leaves and roots, Ovary-all stages and Anther-all stages) as generated by GenEx software. The x-axis shows genes, whereas the y-axis shows Cp values. **a.** B-2S and **b.** B-12-9.

For each B-2S and B-12-9 sample, specific cDNA (leaf, root, anther and ovary) standard curves were generated to measure primer efficiency. The amplification efficiency for primers, calculated by the Light Cycler 4.8 software, ranged from 1.85 ± 0.03 (92.5%) to 2.05 ± 0.05 (102.5%) (Table 2) and were considered appropriate for qRT-PCR [44]. An optimized primer concentration of 300 nM for all genes produced the lowest Cp value (calculated using absolute quantitation *via* second-derivative method). No-RT and no-template controls either did not generate any Cp or the difference in Cp between test and control samples was more than 10 to 13 cycles apart, values considered to be negligible [45]. The six candidate reference genes displayed a relatively wide range of mean Cp values from 17.79 (EF1alpha) to 24.9 (TUBA). The Cp distribution data for B-2S and B-12-9, for all samples (vegetative, ovary-all stages and anther-all stages) are shown in Figure 2a and 2b, respectively. In both B-2S and B-12-9, EF1alpha showed the highest expression whereas TUBA showed the lowest expression level. Studying expression levels of candidate reference genes will allow us to choose reference genes with similar expression patterns to that of the tested genes for future work.

In order to minimize bias introduced by the reference gene validation approach, two programs with different algorithms, GeNorm and NormFinder, were used for measuring gene expression stability. GeNorm software assumes that none of the tested genes being analyzed are co-regulated [46]. The stability measure provided by GeNorm (M-value) is the mean pairwise variation between a gene and all other tested candidates, and hence a pair of highly co-regulated genes could be eliminated during the selection if they show high inter-sample variability [28]. Genes with the lowest M-value are considered most stable. M-values below 0.5 indicate good measure of stability [39,46]. We also tested the NormFinder software since it is less sensitive to co-regulation [28]. NormFinder relies on a ‘model-based approach’; it determines expression stability of candidate reference genes by comparing the expression variation between and within groups and then combines both results in a stability value for each tested gene [47]. For NormFinder, the gene with the lowest standard deviation (SD) will be top ranked [28,32]. NormFinder software was found to be more robust with larger sample sizes [48]. Differences in gene ranking between different stability software have been reported [20].

In order to measure expression stability of genes across organs and stages, the Cp data in the Microsoft Excel file was transported into GenEx software and analysis was performed *via* GeNorm (as described by Vandesompele *et al.*[31]) and NormFinder (as described by Anderson *et al.*[32]). As analysis of gene expression in apomictic development requires the comparison between sexual and apomictic plants, which are both genetically and developmentally different, we analyzed B-2S and B-12-9 tissue types separately. In order to monitor if the developmental stages of ovaries and anthers influence the reference gene stability, organ and/or developmental stage specific analysis was performed. Each sample set was first subjected to GeNorm analysis and all genes with an M-value ≤ 0.5 are reported (Table 3). The GeNorm results were further confirmed using NormFinder. Since there is no cut-off value for standard deviation, SD (stability value of NormFinder), the criterion for genes to be considered stable was that the genes confirmed as stable by GeNorm should be among the top four ranking in NormFinder. The result of NormFinder analysis is shown in Table 3. Although results in Table 3 show EIF4A as the most stable gene across all tissues of B-2S and B-12-9, there were tissue specific changes with respect to gene stability, using both GeNorm and NormFinder (Table 3).

**Table 3:** Tissue specific reference gene stability analyses of B-2S (sex) and B-12-9 (apo) using GeNorm and NormFinder

**Genotype_Tissue type**		**GeNorm - M values**						**NormFinder_Gene ranking (SD values)**					
**Samples**													
		**ACT2**	**EF1alpha**	**EIF4A**	**GAPDH**	**TUBA**	**UBCE**	**ACT2**	**EF1alpha**	**EIF4A**	**GAPDH**	**TUBA**	**UBCE**
Ovary (early/tetrad/DOP)	**Sex**	0.23	0.30	0.19	0.19	0.47	0.37	**1** (0.09)	**4** (0.34)	**3** (0.25)	**2** (0.13)	**6** (0.68)	**5** (0.50)
	**Apo**	0.15	0.15	0.37	0.30	Oc	0.24	**1** (0.07)	**1** (0.07)	**4** (0.48)	**2** (0.22)	**5** (0.87)	3 (0.38)
Ovary (early/tetrad/DOP/DOP + 5)	**Sex**	0.44	Oc	0.16	0.16	Oc	0.39	**4** (0.53)	**6** (1.06)	**1** (0.07)	**2** (0.08)	**5** (0.64)	**3** (0.39)
	**Apo**	0.20	0.20	0.32	0.27	Oc	0.38	**2** (0.14)	**1** (0.10)	**4** (0.40)	**3** (0.18)	**6** (0.74)	**5** (0.48)
Anther (early/tetrad/DOP)	**Sex**	Oc^1^	Oc	0.47	0.47	Oc	Oc	**5** (1.20)	**3** (0.53)	**2** (0.45)	**1** (0.10)	**5** (1.20)	**4** (0.85)
	**Apo**	Oc	0.43	0.27	0.36	Oc	0.27	**6** (0.82)	**4** (0.64)	**2** (0.19)	**3** (0.63)	**5** (0.72)	**1** (0.13)
All tissues	**Sex**	Oc	Oc	0.38	0.38	Oc	Oc	**4** (0.80)	**5** (0.97)	**1** (0.20)	**2** (0.40)	**6** (1.10)	**3** (0.60)
	**Apo**	Oc	Oc	0.46	Oc	Oc	Oc	**3** (0.84)	**4** (0.86)	**1** (0.50)	**2** (0.60)	**6** (0.95)	**5** (0.90)

GeNorm stability value is represented by M value (the selected cut off for M value is ≤0.5). NormFinder stability value is represented by SD (standard deviation). There is no cut-off for SD, hence the ranking of genes based on the SD value is used as a measure to evaluate stability (1 stands for most stable, 6 is least stable). The ovary and anther stages are as follows: early stands for early premeiosis, tetrad, DOP for day of pollination, and DOP + 5 is 5 days after day of pollination.

^**1**^Oc refers to value outside the cut-off range.

**Figure 3:**
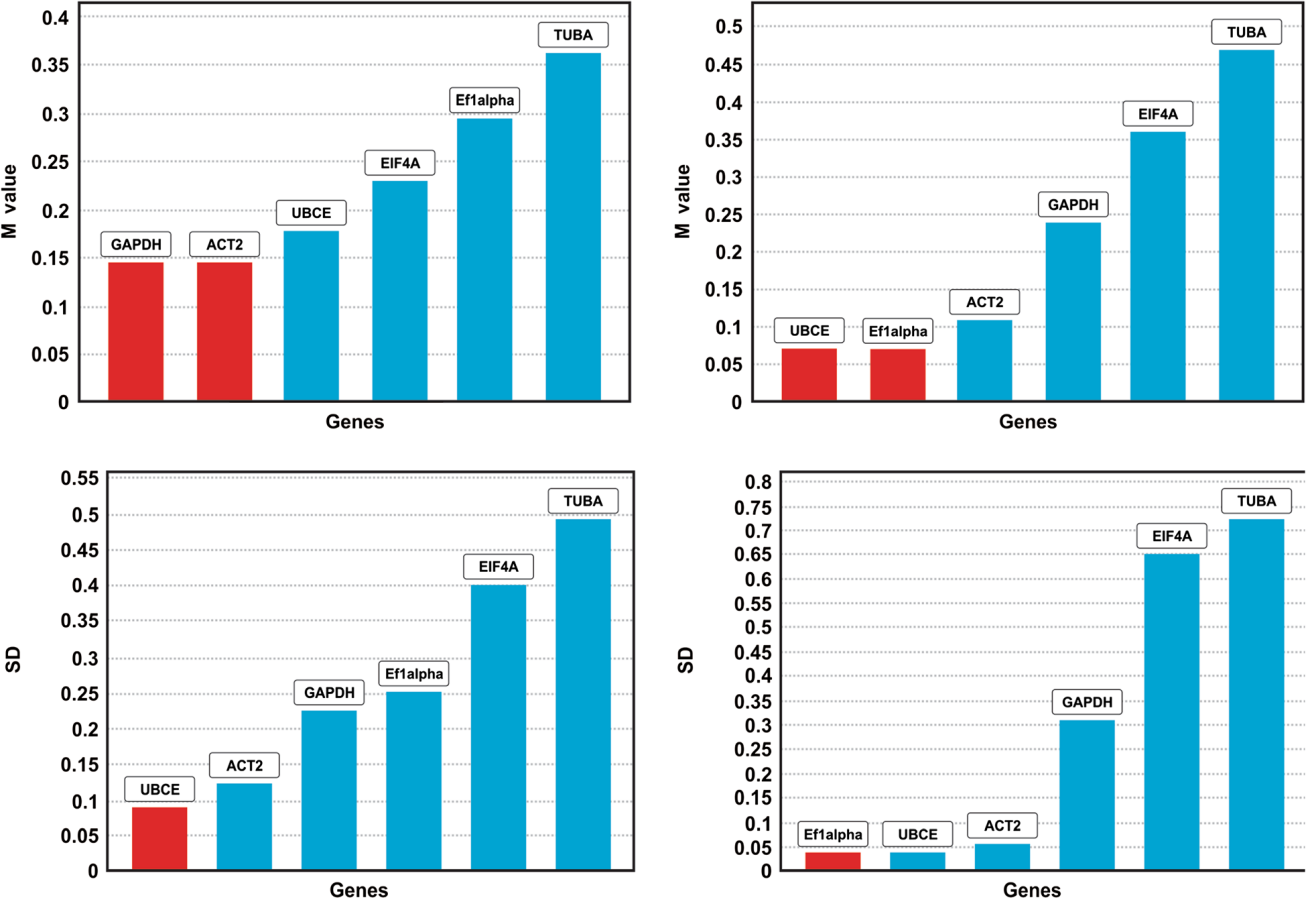
**The GeNorm (I) and NormFinder (II) stability values for B-2S (a) and B-12-9 (b) ovaries at early/tetrad stages.** Boxes in red indicate the most stable genes or gene pairs. SD – standard deviation.

Given our specific interest in ovary development and comparison of gene expression between sexual and apomictic genotypes (in our case B-2S and B-12-9), a detailed analysis of ovary samples of B-2S and B-12-9, delineating groupings of developmental stages, showed very similar results between GeNorm and NormFinder (Table 3). Except for TUBA, all tested reference genes (M-value below 0.5) could be used for developmental grouping up to DOP for sexual vs. apomictic gene comparisons. The addition of the developmental stage DOP + 5, excluded EF1alpha and TUBA as good candidate reference genes. As genes involved in the apomeiosis pathway should be triggered towards early ovary developmental stages (early premeiosis and tetrad), a detailed analysis of early-stage B-2S and B-12-9 ovaries using GeNorm graph, as plotted by GenEx (Figure 3) shows that all genes had an M-value below 0.5. Both GeNorm and NormFinder results for B-2S and B-12-9 showed that the combination of EF1alpha, ACT2, UBCE and GAPDH occupied the most stable positions in the graph.

A second independent assay of B-2S and B-12-9 ovary samples was performed using two independent biological replicates, with samples collected at the same developmental stages (Table 4). Unlike the first analysis, TUBA qualified up to the DOP stage as stable in GeNorm (M <0.5) but was still among the lowest rankings in NormFinder. In the second analysis, EF1alpha was considered stable at DOP + 5. Both analyses of ovaries including DOP + 5 identified TUBA to be unstable in at least one genotype (as per GeNorm). The reference genes EIF4A, GAPDH, UBCE and ACT2 were found to be stable in both first (Table 3) and second (Table 4) analyses of DOP as well as DOP + 5 ovary stages. All tested reference genes qualified for GeNorm stability for early + tetrad ovary tissue in the second analysis (data not shown).

**Table 4:** Ovary (second independent assay) reference gene stability analyses of B-2S (sex) and B-12-9 (apo) using GeNorm and NormFinder

**Genotype_Tissue type**		**GeNorm - M values**						**NormFinder_Gene ranking (SD values)**					
**Samples**													
		**ACT2**	**EF1alpha**	**EIF4A**	**GAPDH**	**TUBA**	**UBCE**	**ACT2**	**EF1alpha**	**EIF4A**	**GAPDH**	**TUBA**	**UBCE**
Ovary (early/tetrad/DOP)	**Sex**	0.19	0.10	0.09	0.03	0.16	0.03	**5** (0.24)	**1** (0.01)	**1** (0.01)	**2** (0.13)	**4** (0.23)	**3** (0.15)
	**Apo**	0.30	0.26	0.33	0.39	0.26	0.45	**4** (0.32)	**2** (0.25)	**1** (0.12)	**3** (0.30)	**5** (0.44)	**6** (0.54)
Ovary (early/tetrad/DOP/DOP + 5)	**Sex**	0.24	0.16	0.09	0.06	0.29	0.06	**4** (0.29)	**5** (0.31)	**1** (0.05)	**2** (0.07)	**6** (0.35)	**3** (0.12)
	**Apo**	0.30	0.30	0.32	0.35	Oc^1^	0.40	**3** (0.23)	**2** (0.16)	**1** (0.10)	**4** (0.28)	**6** (0.81)	**5** (0.56)

GeNorm stability value is represented by M value (the selected cut off for M value is ≤0.5). NormFinder stability value is represented by SD (standard deviation). There is no cut-off for SD, hence the ranking of genes based on the SD value is used as a measure to evaluate stability (1 stands for most stable, 6 is least stable). The ovary stages are as follows: early stands for early premeiosis, tetrad, DOP for day of pollination, and DOP + 5 is 5 days after day of pollination.

^**1**^Oc refers to value outside the cut-off range.

## Conclusion

Our data present the first in-depth analysis of reference gene stability validation in *C. ciliaris*, a tetraploid and important model for understanding apomixis in grasses. Using both apomictic and sexual accessions of *C. ciliaris* and different organs/developmental stages, we were able to understand the reference gene stability specific to each organ, stage, or mode of reproduction. Our analysis used six reference genes based on their previous use in other plant species. These genes were analyzed in 18 samples, from both apomictic and sexual plants. All- tissue analysis using GeNorm and NormFinder found EIF4A as the most stable gene. There was tight correlation between analyses with GeNorm and NormFinder, as genes detected stable by GeNorm occupied top gene rankings in NormFinder analysis.

Detailed analyses of ovary tissue in two independent assays, confirmed stability of genes in specific ovary stages that could be used in both B-2S and B-12-9, irrespective of the mode of reproduction. Based on our results, all tested reference genes were found to be stable (as per GeNorm, M-value below 0.5) for early to tetrad stage ovary development between the two species, while ACT2, UBCE, GAPDH and/or EIF4A would be most suitable for ovary stages up to DOP and DOP + 5. These reference gene expression and stability analyses will provide an important guideline for our future study involving apomixis-related gene expression studies *via* qRT-PCR in *C. ciliaris* or other related grasses.

## Competing interests

The authors declare that they have no competing interests.

## Authors’ contributions

BS led the experiments and drafted the manuscript. JAC and POA conceived the experiments, participated in experimental design, and finalized the manuscript. All authors read and approved the final manuscript.
